# Survival, gene and metabolite responses of *Litoria verreauxii alpina* frogs to fungal disease chytridiomycosis

**DOI:** 10.1038/sdata.2018.33

**Published:** 2018-03-06

**Authors:** Laura F. Grogan, Jason Mulvenna, Joel P. A. Gummer, Ben C. Scheele, Lee Berger, Scott D. Cashins, Michael S. McFadden, Peter Harlow, David A. Hunter, Robert D. Trengove, Lee F. Skerratt

**Affiliations:** 1 Griffith Wildlife Disease Ecology Group, Environmental Futures Research Institute and School of Environment, Griffith University, Nathan, Queensland 4111, Australia; 2 One Health Research Group, College of Public Health, Medical and Veterinary Sciences, James Cook University, Angus Smith Drive, Townsville, Queensland 4811, Australia; 3 Genetics and Computational Biology, QIMR Berghofer Medical Research Institute, 300 Herston Road, Brisbane, Queensland 4006, Australia; 4 School of Biomedical Sciences, The University of Queensland, Brisbane, Queensland 4072, Australia; 5 Separation Science and Metabolomics Laboratory, Murdoch University, Perth, Western Australia 6150, Australia; 6 Metabolomics Australia, Murdoch University Node, Murdoch University, Perth, Western Australia 6150, Australia; 7 Fenner School of Environment and Society, Australian National University, Canberra, Australian Capital Territory 2601, Australia; 8 Taronga Conservation Society Australia, Bradleys Head Road, Mosman, New South Wales 2088, Australia; 9 Ecosystems and Threatened Species, South West Region, NSW Office of Environment and Heritage, Albury, New South Wales 2640, Australia

**Keywords:** Ecological epidemiology, Transcriptomics, Conservation biology, Metabolomics, Fungal infection

## Abstract

The fungal skin disease chytridiomycosis has caused the devastating decline and extinction of hundreds of amphibian species globally, yet the potential for evolving resistance, and the underlying pathophysiological mechanisms remain poorly understood. We exposed 406 naïve, captive-raised alpine tree frogs (*Litoria verreauxii alpina*) from multiple populations (one evolutionarily naïve to chytridiomycosis) to the aetiological agent *Batrachochytrium dendrobatidis* in two concurrent and controlled infection experiments. We investigated (A) survival outcomes and clinical pathogen burdens between populations and clutches, and (B) individual host tissue responses to chytridiomycosis. Here we present multiple interrelated datasets associated with these exposure experiments, including animal signalment, survival and pathogen burden of 355 animals from Experiment A, and the following datasets related to 61 animals from Experiment B: animal signalment and pathogen burden; raw RNA-Seq reads from skin, liver and spleen tissues; *de novo* assembled transcriptomes for each tissue type; raw gene expression data; annotation data for each gene; and raw metabolite expression data from skin and liver tissues. These data provide an extensive baseline for future analyses.

## Background & Summary

Over one third of all amphibian species around the world are threatened with extinction^1^. One of the main causes of declines is the spread of the lethal skin disease chytridiomycosis, caused by the fungal pathogen *Batrachochytrium dendrobatidis* (Bd)^2–4^. Despite considerable research in recent decades, methods for disease mitigation *in situ* are still in their infancy^5^. Accelerated evolution of disease resistance via marker-assisted selection may provide a long-term management solution for species at continued risk of decline^6,7^. Such evolution of disease resistance has been demonstrated in natural disease systems^8,9^, and marker-assisted selection has been widely utilized in domestic animals and agriculture^10,11^.

The mechanisms underpinning chytridiomycosis pathophysiology and the potential for the evolution of host resistance are currently poorly understood^12^. This is partly because as yet there is no clear, controlled example of the evolution of host resistance to chytridiomycosis which would enable investigation of evolving mechanisms of resistance. One way to test for evolution of resistance without undertaking logistically infeasible longitudinal studies is by comparing the survival of experimentally challenged frogs between exposed populations and geographically-related Bd-naïve populations. Geographic proximity acts to control for other evolutionary forces by providing ecological similarity other than the presence of chytridiomycosis.

**Figure 1:**
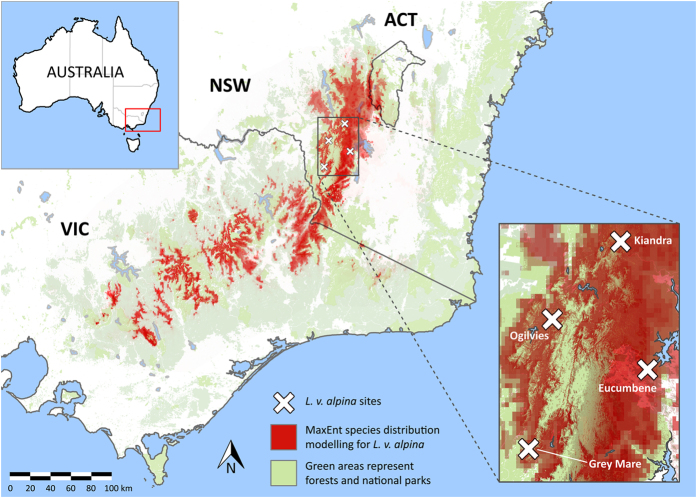
Map demonstrating location of the four source populations of *L. v. alpina* used in this study. Inset shows greater detail. Red shading represents the predicted distribution range of *L. v. alpina*, using species distribution modelling algorithm MaxEnt and species presence data sourced from the Atlas of Living Australia (ALA) and filtered according to expert knowledge of subspecies distributions^41–45^. Green shading represents state forests and national parks. The effects of chytridiomycosis on the distribution of *L. v. alpina* have not been incorporated.

The endangered alpine tree frog (*Litoria verreauxii alpina*), an endangered and highly Bd-susceptible subspecies endemic to the sub-alpine regions of Victoria and New South Wales in south-eastern Australia provided us with a unique opportunity to explore this question of the potential for evolution of host resistance. Throughout its range, *L. v. alpina* experienced major declines from the early 1980 s when Bd is believed to have arrived^13^, resulting in the extirpation of most known populations^14,15^. However, we were aware of one population (Grey Mare in Kosciuszko National Park, Australia; Fig. 1) that by virtue of relative geographic isolation had hitherto eluded disease emergence.

We thus collected multiple clutches of eggs from this infection-naïve population in 2010, plus three other exposed populations (first exposed to Bd approximately 26 years earlier based on unpublished reports of declines) and raised the frogs in captivity. The naïve population, Grey Mare, remained uninfected until late in 2012, when the arrival of Bd at this site had a catastrophic effect, with few frogs observed since (B. Scheele unpublished data). A small number of remaining *L. v. alpina* populations now persist despite high ongoing annual adult mortality associated with chytridiomycosis^13,15^. Multiple studies have been performed on *L. v. alpina* at these study sites, providing a comprehensive background of ecological and disease dynamic information^13,15–20^.

In this study we examined clinical host susceptibility (survival and infection intensity), and underlying gene expression and metabolomics responses of key host tissues to compare the immunology and pathophysiology of infection between populations. Our aim was to investigate whether persisting long-exposed populations may have evolved disease resistance compared with the naïve population, and to characterize potentially resistant phenotypes. We performed two related experiments on a total of 406 adult *L. v. alpina* via controlled laboratory Bd-exposure. The specific aims of our experiments and data collection were to (1) examine associations between survival, infection burden, and tissue immune and physiologic responses (via measurement of gene expression and metabolite accumulation) within individuals, clutches, and populations of frogs, (2) compare population responses between naïve and long-exposed populations, and (3) compare infection responses at various times post exposure, with a focus on the subclinical infection stage.

**Figure 2:**
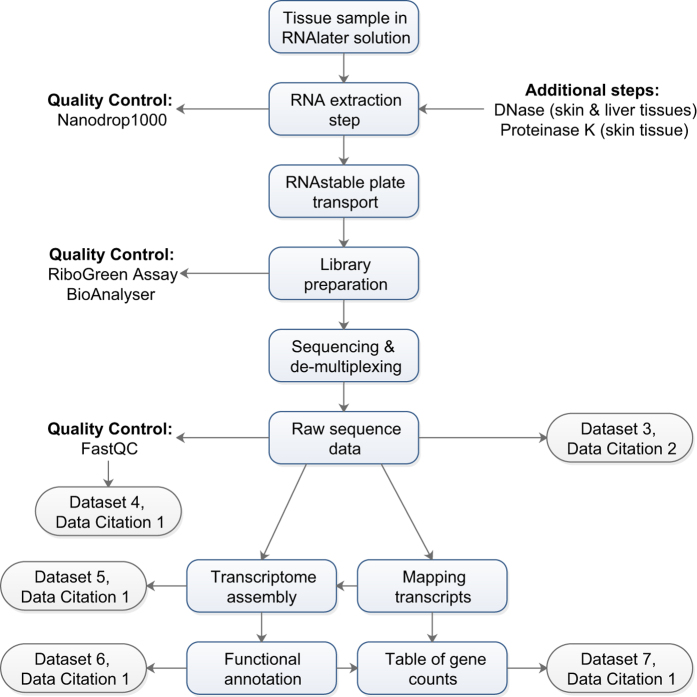
Schematic overview of the transcriptomic analyses performed in the study, and the corresponding quality control measures and data outputs. Three tissues were collected from each frog (n=61) for transcriptomic analysis, yielding a total of n=181 samples (2 missing data). All tissue samples underwent RNA extraction, however skin and liver samples had their total RNA extracted with 5-Prime PerfectPure kit and were subject to a DNase step, skin samples were subject to a Proteinase K step, and spleen samples had their RNA extracted with Qiagen RNeasy minikit. All RNA samples were subject to quality control steps, library preparation and sequencing, producing raw sequence data for each sample. Tissue-specific transcriptomes were assembled *de novo* with Trinity^34^ and functionally annotated with BLAST2GO^36^ (with inbuilt quality control measures, including minimum e-value and percentage similarity), and henceforth all sample data were aligned and annotated based on these.

The specific data we collected included animal signalment, survival, pathogen burden, raw RNA-Seq reads from skin, liver and spleen tissues, *de novo* assembled transcriptomes for each tissue type, raw gene expression data, annotation data for each gene, and raw metabolite expression data from skin and liver tissues. Tables 1 and 2 provide a quantitative overview of the numbers of frogs, their source populations, clutches and respective treatments in the exposure experiment. Table 3 (available online only) provides summary descriptions of the data associated with this manuscript. Figure 2 provides a schematic overview of the steps involved in transcriptomic analysis and the corresponding data outputs. These data have already been used to examine gene associations with survival^16^, differential gene expression^21^ and metabolite accumulation (L. Grogan unpublished data). Additional analyses could include (1) the investigation of specific genes or gene groups of interest, identified using sequence information provided by transcriptome assembly, (2) the examination of gene and isoform variation between populations, and (3) the determination of novel or common mechanisms of resistance/susceptibility amongst other well characterised disease systems.

## Methods

### Animals were sourced as infection-naïve eggs from four wild populations

Up to four clutches of alpine tree frog eggs (*Litoria verreauxii alpina*) with unknown but possible multiple paternity were collected by D. Hunter in 2010 from each of four ecologically similar but geographically distinct wild populations from around Kosciuszko National Park, New South Wales, Australia, in accordance with Scientific Licence number S12848 (total of 15 clutches). The source populations are hereafter designated Kiandra (35.872°S 148.500°E 1356 m above sea level [asl]), Eucumbene (36.152°S 148.563°E 1451 m asl), Grey Mare (36.317°S 148.260°E 1525 m asl), and Ogilvies (36.036°S 148.322°E 1307 m asl) (Fig. 1). Detailed descriptions of the sites can be found in Scheele et al. (2015)^15^. Three of the four populations (Kiandra, Eucumbene and Ogilvies) had a multi-generational history (over two decades) of exposure to the fungal pathogen Bd^13,14^. The remaining population (Grey Mare) was at the time evolutionarily naïve to the pathogen due to relative geographic isolation, until the pathogen’s emergence there in 2012 (B. Scheele unpublished data).

### Captive husbandry was performed under quarantine conditions

Animals were captively-reared in clutch groups at Taronga Zoo, Sydney, Australia, until approximately eight months post-metamorphosis (in 2011). Experienced animal handlers followed routine rearing protocols under quarantine conditions in accordance with guidelines approved by Taronga Conservation Society Animal Ethics Committee (4c/01/10). Adult frogs were transferred to individual enclosures several weeks prior to commencement of the infection exposure experiment to allow for acclimatization to enclosures and conditions. From this time, frogs were maintained at constant 19 °C (±2 °C) in individual plastic containers with permeable gauze lids, pebble substrate, and water drainage holes down one end of the tub. Enclosures were placed at a slight angle (10°) to facilitate water drainage and provide wet and dry areas. Frogs were fed multivitamin and calcium-dusted crickets alternately twice weekly *ad libitum*. Individual frogs were observed daily by an experienced animal handler and veterinarian for examination of health status and clinical signs of disease. A small hand-held hose was used to flush tubs daily via the gauze top of the tub. Tubs were flushed for at least 15 s with filtered water until the water ran clear and debris was removed.

### Experimental design involved blinded, randomized and controlled treatment groups

Experimental protocols involving animals were carried out in accordance with the approved guidelines and protocols under permits issued by James Cook University (A1408) and Taronga Conservation Society (4c/01/10) Animal Ethics Committees. From among the captively-reared *L. v. alpina*, a total of 406 chytridiomycosis-naïve adult frogs were prepared for the exposure experiment. The number of frogs available for utilization was subject to original clutch size and natural attrition during growth and development. An unexposed negative control group was randomly assigned to control for experimental effects, and operators were blinded to treatment groups. We maximized the sample size of exposed frogs for experimental purposes by randomly selecting up to 20 frogs from each block (blocks consisted of specific clutches, A-D, within the four populations) to be exposed to Bd. Some clutches were of insufficient sample size to permit the allocation of negative control individuals (Eucumbene clutch B, Grey Mare clutches A and D, Ogilvies clutches A and C).

We needed to run two concurrent experiments under identical conditions in order to link survival results (Experiment A) with tissue-level responses (Experiment B). This experimental design was necessary because animals had to be sacrificed during the course of infection in Experiment B in order to obtain tissues. Our experimental design accommodated for this purpose by using siblings across both experiments that were collected from the same wild egg clutches (with possible multiple-paternity).

#### Experiment A–Survival experiment

Experiment A utilized 355 frogs in a clinical survival experiment and involved random allocation of frogs from all populations and clutches, according to a controlled, randomized, stratified block design outlined in Table 1.

#### Experiment B–Tissue response experiment

Experiment B utilized 51 frogs independent of those in Experiment A, plus an additional 10 frogs from Experiment A that were sampled upon euthanasia when showing clinical signs of chytridiomycosis. The dual use of the 10 frogs from Experiment A allowed us to reduce the total number of frogs used for ethical reasons while improving statistical power. The purpose of Experiment B was to investigate underlying immune and pathophysiological responses to infection. Frogs were randomly selected from specific clutches (corresponding to clutches also used in the survival experiment) and humanely euthanized for tissue sampling at various time points post-exposure. This experiment involved 18 frogs from each of Eucumbene (Clutch D) and Kiandra (Clutch B) populations (including 6 negative control animals from each population), and 15 frogs from Grey Mare (Clutch B) (including 3 negative control animals; Table 2), plus the additional 10 frogs from Experiment A (4 frogs from Grey Mare, and 2 each from the remaining 3 populations). Our study design resulted in 3 to 4 biological replicates per group at the lowest level of comparison (comparing gene and metabolite expression from each population and sampling time post exposure; as commonly utilized in transcriptomics studies), and higher numbers in the pooled group comparisons (comparing populations, or sampling times post exposure). In order to examine for batch effects, we sampled control frogs from across all sampling sessions rather than within a single session. Initial clutch sizes were limiting in some cases, leading to a lower overall number of frogs available (particularly regarding the number of control frogs from Grey Mare).

### Frogs were exposed to Bd or sham-exposed to DSS

A Bd strain (AbercrombieNP-L.booroolongensis-09-LB-P7), isolated in May 2009 from a healthy adult frog and minimally passaged (from Abercrombie River National Park, a site roughly 230 km north-east of the study sites), was used for inoculations on 20th September, 2011. All Australian isolates examined thus far have been identified to belong to the hypervirulent global panzootic lineage (Bd-GPL^6,22^). The strain was maintained and infectious zoospores collected and counted as described previously^23^. In brief, we cultured Bd at 20 °C on mTGhL agar plates (8 g L^−1^ tryptone, 2 g L^−1^ gelatin hydrolysate, 4 g L^−1^ lactose, 10 g L^−1^ agar, with the addition of 200 mg L^−1^ penicillin-G and 200-500 mg L^−1^ streptomycin sulfate) before flooding plates with 10 mL dilute salts solution (DSS: 1.0 mMol KH_2_PO_4_, 0.2 mMol CaCl_2_.H_2_O, 0.1 mMol MgCl_2_.2H_2_O) for 20 min. Zoospore concentration was determined using a haemocytometer and dilutions were adjusted for consistency.

Frogs were confirmed negative to Bd (via qPCR, see below) prior to the commencement of the exposure experiment, and assigned an identification number (Lva001-Lva406) randomly with respect to population, clutch and treatment group. Two frogs died during the acclimatization period pre-exposure (Lva209 and Lva303), and another frog died post-exposure from a condition unrelated to chytridiomycosis (anasarca and cloacal prolapse; Lva162). Data from the former two frogs is not included, leaving a total of 404 experimental animals (details in Table 1). A total of 278 frogs from Experiment A (including the 10 crossed-over to Experiment B), and 36 frogs from Experiment B were individually exposed to 750,000 zoospores in 25 mL DSS, with enclosures held flat without pebble substrate for 18 h. Following exposure, enclosures were held horizontally for a further two days, however, the water was replaced daily, before having pebbles added and being placed again on a mild angle (10°) for ease of drainage. Seventy-five negative control frogs from Experiment A and 15 from Experiment B were subject to identical conditions, but were instead sham-exposed with only DSS.

Animal discomfort was minimized, and individual frogs were observed daily by an experienced animal handler and/or veterinarian for health status. If clinical signs of chytridiomycosis infection were detected (dullness, lethargy, peripheral erythema and increased skin shedding), the frog was immediately humanely euthanized using an overdose of a concentrated buffered anaesthetic agent tricaine methanesulfonate (MS-222). Survival data (date of death), measurement data (mass, snout-urostyle length, sex), and skin swabs for Bd infection intensity (ascertained via qPCR as described below) were collected for frogs from Experiment A (Dataset 1; Data Citation 1).

### Skin, liver and spleen tissues were sampled at specific times post exposure (Experiment B)

Three sampling sessions were performed at 4, 8 and 14 days post exposure (DPE; corresponding with subclinical infections) using a randomized block design (each session sampled a total of 17 animals, including control frogs (stratified by populations of origin; see Table 2 for experimental design). In addition to the 51 frogs already described, 10 exposed frogs were obtained from Experiment A and sampled between 28 and 30 DPE when they began to exhibit clinical signs of chytridiomycosis and were hence in the terminal stages of disease. These additional frogs included two from each of Kiandra, Eucumbene, and Ogilvies populations, and four frogs from Grey Mare. Immediately prior to humane euthanasia (via rapid double pithing, to avoid possible effects of MS-222 on tissue physiology), frogs were examined, weighed, had their snout-urostyle length measured, and were skin swabbed (as described below) to confirm infection status and to quantify Bd infection intensity via qPCR (Dataset 2; Data Citation 1).

Immediately following euthanasia of each frog, a post-mortem examination via ventral midline coeliotomy was performed for collection of tissues for subsequent analyses. Tissues collected included (1) ventral abdominal skin and liver that were immediately transferred to 500 μL 100% methanol and stored at −80 °C, and (2) ventral abdominal and thigh skin, spleen and liver that were immediately transferred to RNAlater (Qiagen, Australia) and refrigerated overnight at 4 °C before being stored longer-term at -80 °C. The ventral abdominal and thigh skin of frogs are common sites for Bd infection^2,24^. The spleen is the main peripheral lymphoid or immunological organ of frogs^25^. The liver is an important organ for many physiological functions including several associated with both immunity and pathogenesis of infectious disease (such as immune surveillance, detoxification of antigens, removal of debris, breakdown of the products of apoptosis, and assimilation of energy^26^). Sex was ascertained via examination of coelomic reproductive organs and forelimb nuptial pads on males (sexual dimorphism in frogs of this age was not consistently expressed).

### Swabs were collected weekly and pre-euthanasia to determine infection intensities via qPCR (Experiment A)

Bd infection intensity data on individual frogs (in zoospore equivalents, ZSE) was obtained by first collecting skin swabs. Swabbing was conducted pre-euthanasia for every frog, as well as weekly for frogs from Experiment A from week two post-exposure onwards, including on the final day of the experiment for all surviving frogs (15th December, 2011; total experimental length 86 days). Swabbing was conducted under quarantine conditions using new gloves and a new sterile dry swab per frog (MW 100-100; Medical Wire and Equipment, Bath, UK). Swabs were rotated continuously while being drawn along the palmar and plantar surfaces of each of the fore and hind-feet four times, as well as both sides of the ventral abdomen. Swabs were sealed individually and stored dry at 4 °C, or frozen longer-term at −20 °C. A selection of collected swabs from weekly sampling sessions, as well as all pre-euthanasia swabs, were analyzed for Bd DNA in triplicate with the TaqMan real-time qPCR protocol^27,28^, and each run included an internal positive control. Samples that recorded only one or two of the three wells as a low positive were considered positive to maximise diagnostic sensitivity^29^.

### Transcriptomics methods

#### RNA extractions

Total RNA was isolated from skin, liver and spleen tissue samples that had been stored in RNAlater following the manufacturer’s protocol for 5-Prime PerfectPure RNA Tissue kits (Eppendorf-5 Prime, Boulder, CO, USA) for liver and skin samples and Qiagen RNeasy mini kits for spleen samples (the spleens were considerably smaller in volume and required a more sensitive method). Tissues were first removed from RNAlater and then lysed on ice using a rotor-stator homogenizer. Liver and skin lysates were treated with DNase to remove genomic DNA, and skin lysates were subject to an additional proteinase K step to digest keratin, remove excess proteins, and inactivate nucleases. Pure total RNA was eluted in nuclease-free water. RNA quantity was determined with a Nanodrop 1000 spectrophotometer, the absorption ratios 260/280 (~2.0) and 260/230 (2.0-2.2) were assessed, and the spectral pattern was evaluated in order to determine RNA integrity and purity. RNAstable plates (Biomatrica, California USA) were used to ship total RNA samples dry and at room temperature to Minnesota BioMedical Genomics Centre, USA.

#### RNA-Seq using the Illumina HiSeq 2000 platform

Total RNA samples were reconstituted with nuclease-free water, quantified with a fluorimetric RiboGreen assay, and had their quality assessed with capillary electrophoresis (Agilent BioAnalyzer 2100; Agilent, Santa Clara, CA, USA). Samples containing >1 ug total RNA and having an RNA Integrity Number (RIN)>8 passed quality control. Copy DNA Illumina sequencing libraries were created from each of the samples following manufacturer specifications (Illumina Truseq RNA Sample Preparation Kit), and up to 12 samples were individually indexed by the ligation of adaptors for multiplexing on each flow cell lane. Indexed libraries were gel-size selected to 320 bp +/− 5%, and hybridized to a paired end flow cell, before being clonally amplified by bridge amplification, and sequenced with the Illumina HiSeq 2000 (Illumina, San Diego, CA). Base call data for paired end reads from each sample were analyzed and de-multiplexed with CASAVA software 1.8.2 (Illumina, San Diego, CA) generating .fastq files for each sample (Data Citation 2).

#### Transcriptome assembly, annotation, and sample gene abundance quantification

The 100 bp paired end read sequences from each sample were first examined for read quality using FastQC as described under Technical Validation below (Dataset 4; Data Citation 1). Trimmomatic 0.30 (ref. 30) was run in paired end mode to trim TruSeq adapter sequences, crop Illumina's random hexamers^31^, perform sliding window trimming (window size 4, required quality 15), trim low quality bases from each end of reads (required quality 3), and ensure that the minimum length of resultant reads was at least 75 bases. Widowed reads were retained as unpaired forward and reverse read files. We performed digital normalization to reduce overall data set size and remove read redundancy using DigiNorm^32^ with the -p flag for the paired read files. We then used Bowtie^33^ to remove reads aligning to the Bd genome. The remaining reads were then used to generate *de novo* assemblies for each tissue type (with Trinity^34^) which were filtered to remove erroneous contigs (see Technical Validation section). Protein coding regions in the assembled transcripts were then identified using TransDecoder (Trinity) (Dataset 5; Data Citation 1).

The resulting transcript sequences were functionally annotated using BLAST2GO^35,36^ against an in-house anuran database consisting of the Amphibia subset of the National Center for Biotechnology Information (NCBI; http://www.ncbi.nlm.nih.gov) non-redundant protein database. Functional annotation data from the Gene Ontology [GO] consortium, Enzyme Code and Inter Pro databases are contained in Dataset 6 [Data Citation 1]. The R package RSEM^37^ was then used to quantify the abundance of genes and isoforms in individual frog tissue samples in combination with the above-described tissue-specific assembled transcriptomes, generating tables of transcript count data (Dataset 7; Data Citation 1).

### Metabolomics methods

#### Metabolite isolation

The harvested tissue was transferred from methanol to a pre-chilled (dry ice) 2 ml cryo-tube. Each sample tube was then submerged in liquid nitrogen and the sample dried by lyophilisation in a LABCONCO Freezone 2.5 Plus freeze-dryer (Labconco Corp., USA). The dried samples were homogenized by adding approximately 30 x 1.4 mm ceramic beads (Precellys, France) to each cryo-tube, followed by six 20 s rounds of vigorous agitation at 6,500 *rpm* in a Precellys 24 lysis cryo-mill (Bertin technologies, France). The metabolites were extracted by the addition of 500 μL of ice cold methanol to the homogenate, a further 20 s of vigorous agitation as already described, and 15 min mixing at 1,400 rpm at 4 °C in an Eppendorf Thermomixer (Eppendorf, USA). Cell debris were collected by centrifugation at 16,100 *g* for 30 min at 4 °C, and the supernatant containing metabolites transferred to a fresh microcentrifuge tube. The extraction process was repeated three times with a second 500 μL of ice cold methanol, and finally using 300 μL of water. The supernatants were combined, and any remaining cell debris and/or precipitate collected by centrifuge as already described. The extracts were dried in preparation for derivatisation ahead of GC-MS analysis as described by Gummer, et al.^38^. Briefly, this involved vacuum concentration of the metabolite extract, followed by dilution with 400 μL of LC-MS grade water, snap-freezing using liquid nitrogen and drying by lyophilisation. The entire volume of the skin-recovered metabolite extracts were dried, but only half of the recovered volume for the liver tissues (due to the larger sample weight). Additional metabolite extracts were set aside from surplus skin tissue to create a pooled extract, as later described.

#### Metabolite preparation and analysis

*Trimethylsilylation (TMS) derivatisation of metabolites for GC-MS analysis*. To ensure the amenability of metabolites to GC-MS analysis, the metabolites were derivatised by a combination of oximation (MEOX) and trimethylsilylation (TMS) as previously described^38^. The metabolite lyophilisates were dissolved in 20 μL of pyridine (20 mg mL^−1^ methoxylamine HCl; Sigma-Aldrich) and incubated at 30 °C with agitation at 1,400 *r.p.m.* for two hours, followed by the addition of 40 μL N-Methyl-N-(trimethylsilyl) trifluoroacetamide (MSTFA; Sigma-Aldrich) and incubation at 37 °C with agitation at 650 *r.p.m.* for one hour. The derivatized metabolites were transferred to a 200 μL glass vial insert within a 1.5 mL glass amber vial. Five μL of hexane containing eight n-alkanes; n-decane, n-dodecane, n-pentadecane, n-nonadecane, n-docosane and n-octacosane at 6.25 μg mL^−1^, and n-dotriacontane and n-hexatriacontane at 13.5 μg mL^−1^, were added to each sample prior to GC-MS analysis.

*GC-MS analysis of metabolites*. Derivatised metabolites (1 μl) were analysed by GC-MS (Shimadzu QP2010 Ultra, Kyoto, Japan). The GC was equipped with an Agilent FactorFour VF-5 ms column (30 m x 0.25 mm x 0.25 μm+10 m EZ-Guard). The injection inlet temperature was 230 °C, with an interface temperature of 300 °C, and an ion source temperature of 230 °C. Helium was used as the carrier gas at a flow of between 0.8 and 1.0 mL min^−1^. The inlet pressure was adjusted to elute mannitol (6-TMS) at 30.6 min. The temperature gradient consisted of an initial temperature of 70 °C, increasing at 1 °C per minute for 5 min before increasing to a final temperature of 320 °C at an oven ramp rate of 5.6 °C min^−1^ with a 10 min hold at maximum temperature. Ionisation was by electron ionization (EI) at 70 eV. The MS was operated in scan in the range m/z 40–600, at a scan rate of 5,600 amu sec^−1^. For selected ion monitoring (SIM), ions were scanned at 0.38 s intervals.

#### Metabolite identification and processing

GC-MS data were acquired and analysed using GCMSsolution 2.61 (Shimadzu Corporation, Kyoto, Japan) and AnalyzerPro 2.7.0.0 (Spectral Works, Runcorn, UK). From a preliminary GC-MS analysis of skin and liver metabolites using a scan mode of acquisition, a selective ion monitoring (SIM) method was assembled using characteristic ions from a metabolite MS library and the preliminary data; and the remaining sample set analysed using a simultaneous full scan and SIM mode of acquisition. Chromatograms were deconvoluted by background subtraction of partially co-eluting ions. Relative analyte quantitation was achieved using a characteristic quantifier ion for each analyte [avoiding Si(CH_3_)_3_, m/z 73; characteristic of the silylation reaction]. Ions exceeding the upper dynamic concentration of the GC-MS were not used for quantitation, with lower abundant ions selected as alternative quantifier ions.

Metabolite identities were determined by mass spectral library search using the acquired scan data, using GCMSsolution 2.61 (Shimadzu Corporation, Kyoto, Japan) and AnalyzerPro 2.7.0.0 (Spectral Works, Runcorn, UK). The identities were assigned by comparison to authentic metabolite standards, requiring a similarity index of 80% or higher MS match and a retention index (RI) of±3. The identification of metabolites without match to an available metabolite standard was sought from mass spectral similarity to an external MS library: Massbank, National Institute of Standards and Technology (NIST) or Wiley Registry™, and used for ‘putative’ metabolite assignments. An ‘unknown’ classification was assigned to metabolites that matched the mass spectra of a known metabolite class, with 70-80% similarity, but where the RI criteria did not match to that of the known metabolite standard. Metabolites without a match to known spectra were also deemed ‘unknown’ and named by the analytical characteristics using the prefix ‘unknown’, followed by the observed retention time (RT) and calculated RI. Metabolite accumulation data were normalised using the summed ion signal (total ion chromatogram) (Data Citation 3) (Dataset 9; Data Citation 1).

## Data Records

For this study we deposited nine datasets. The first consists of animal signalment, survival and pathogen infection intensity data from the animals in Experiment A. These data are contained in Dataset 1, as a single comma-separated file (.csv format), entitled ‘Dataset 1 Experiment A results data’ (Data Citation 1). Each row below the header represents an individual frog (n=353, from the original 355 minus two whose data were removed due to mortality pre-exposure), and columns (n=55) contain frog identifier and treatment group information, signalment and measurement data, raw qPCR results, and length of survival data. Full descriptions of the column headers are contained in the associated readme file. Please see the associated metadata records in Supplementary File 1 for full details of study subjects and swab samples collected.

The second dataset consists of animal signalment and pathogen infection intensity data from the animals in Experiment B. These data are contained in Dataset 2, as a single comma-separated file (.csv format), entitled ‘Dataset 2 Experiment B results data’ (Data Citation 1). Each row below the header represents an individual frog (n=61, from the original 51 frogs in this experiment plus the 10 frogs that were obtained from Experiment A and sampled when moribund at 28-30 days post exposure). Columns (n=27) contain frog identifier and treatment group information, signalment and measurement data, and qPCR results data. Full descriptions of the column headers are contained in the associated readme file. Please see the associated metadata records in Supplementary File 1 for full details of study subjects and swab samples collected.

Dataset 3 consists of raw RNA-Seq reads from skin, liver and spleen tissues of each frog sampled as part of Experiment B (n=61). Due to the multiple tissues sampled per frog (and a couple of poor quality samples that were excluded from library preparation) this dataset consists of 362 individual .fastq.gz files, comprising forward (R1) and reverse (R2) reads from paired-end Illumina Hi-Seq2000 sequencing for each of 60 liver, 61 skin, and 60 spleen tissue samples. These data have been submitted to the NCBI Sequence Read Archive and can be found under accession number: SRP096145 (Data Citation 2). Please see the associated metadata records in Supplementary File 1 for full details of study subjects and tissue samples collected.

The fourth dataset consists of a summary of the phred Q-score quality control data accompanying the sequence data from each tissue sample (described above). These data are contained in Dataset 4 as a single comma-separated file (.csv format) (Data Citation 1). Descriptions of the column headers are contained in the associated readme file.

The fifth dataset consists of the *de novo* assembled transcriptomes for each tissue type from frogs in Experiment B (n=61). These data are contained in Dataset 5, as six individual .fasta text files, including both nucleotide sequence assembly files and amino acid sequence assembly files (translated using TransDecoder) for each of the three tissue types (skin, liver and spleen) (Data Citation 1). Descriptions of the sequence identifier lines are contained in the associated readme file.

The sixth dataset consists of functional annotation data (from the Gene Ontology [GO] consortium, Enzyme Code and Inter Pro databases) for each gene assembled, across the three tissue types (skin, liver and spleen). These data are contained in Dataset 6, as three individual comma-separated files (.csv format) (Data Citation 1). Full descriptions of the column headers are contained in the associated readme file.

The seventh dataset contains the raw gene/transcript counts data for each frog tissue sample (Experiment B, n=61) resulting from allocation of sequence reads to genes from the transcriptome assemblies. These data are contained in Dataset 7, as 12 individual tab-separated text files (.txt) and comma-separated files (.csv) (Data Citation 1). The four files for each tissue type (skin, liver and spleen) represent raw (1) counts, (2) TMM.EXPR files, (3) TPM.not_cross_norm files, and (4) normalisation information (as per the standard output from RSEM). The first row of these files (1-3 above) represents frog ID, and the first element of all subsequent rows represents transcript/isoform ID.

The eighth and ninth datasets contain the metabolite accumulation data from skin and liver tissues for each frog from Experiment B (n=61). These data have been scaled by dividing by the summed ion signal (total ion chromatogram). Dataset 8 has been submitted to the MetaboLights data repository (Data Citation 3). An alternative presentation of this data is also contained as a single comma-separated file (.csv format) (Dataset 9; Data Citation 1), with full descriptions of the column headers (and associated analyte data) contained in the associated readme file. Please see the associated metadata records in Supplementary File 1 for full details of study subjects and tissue samples collected for the metabolomics work.

## Technical Validation

### Quality control of the frog exposure experiment

#### Blinded, controlled and stratified randomized design of the frog exposure experiment

We controlled for technical confounding and potential batch effects *a priori* in our experimental design using a variety of standard techniques including (1) blinding operators to treatment groups, (2) including sham-exposed negative control treatment groups of frogs, and (3) employing a stratified random design. At the commencement of the pre-experimental acclimation period, frogs were assigned a unique ID number (eg, Lva018) that was randomized with respect to source population and clutch and distributed randomly among frogs from Experiments A and B. Frogs were then assigned to treatment groups (including control groups) and prospective sampling periods randomly using a stratified block design (ie, frogs were chosen randomly from among source population and clutch groups up to a total number determined *a priori* for each treatment).

#### Physical layout of the frog exposure experiment

Frogs in the exposure experiments were physically placed according to their randomized ID number in rows on treatment shelves, whereby individuals from different clutches, populations, treatments and sampling dates were placed randomly with respect to each other. Both Experiment A and B were run concurrently under identical environmentally-controlled quarantine conditions inside an air-conditioned laboratory with artificial light source and filtered water source.

#### Controlling for potential batch effects due to date of sampling period

In order to control and test for potential batch effects that might be introduced through the multiple sampling dates utilized for Experiment B (for example, associated with differences in environment or technique, or improved efficiency with repeated sampling through time), at each of the three subclinical sampling sessions (4, 8, and 14 days post exposure), we additionally sampled sham-exposed negative control individuals from each population (from the stratified random design). The sample results from these individuals were then able to be compared between sampling sessions to identify batch effects.

### Quality control of the transcriptomics study

#### Total RNA extraction protocol quality control

The extraction of total RNA from frog tissue samples was performed with standard kits using manufacturer protocols according to frog ID number within tissue type. That is, over several days, all liver samples were processed, following frog ID, then all skin samples were processed likewise, followed by all spleen samples. We acknowledge that as a logistical necessity, different brand kits were used to extract total RNA from skin/liver samples as opposed to spleen samples, however this should not impact resulting analyses because the results are analyzed in a tissue-specific manner (and data from different tissues were not intended to be compared due to the vastly different gene expression profiles expected).

#### Total RNA sample quality and quantity measures

All resulting total RNA samples were subject to rigorous quality control prior to library preparation, including (1) analysis of RNA concentration, 260/280 (~ 2.0) and 260/230 (2.0-2.2) absorption ratios via Nanodrop 1000 spectrophotometer, (2) quantification using a fluorimetric Quant-iT RiboGreen RNA assay, and (3) quality assessment using capillary electrophoresis (Agilent Caliper LabChip GX high-throughput BioAnalyzer). To pass quality control samples had to have>1 μg, and the RNA Integrity Number (RIN)>8. In-house results for each sample from the Nanodrop 1000 spectrophotometer are included in the associated metadata record.

#### Quality controls during sequencing

Multiplexing and sequencing via Illumina HiSeq-2000 platform were performed following standard protocols with PhiX Control v3 internal control spiked at 1% into every lane of each flow cell (as recommended by the manufacturer), by the University of Minnesota’s BioMedical Genomics Centre (now known as University of Minnesota Genomics Center (UMGC); http://genomics.umn.edu/). Samples were multiplexed into flow cell lanes by tissue type.

#### FastQC for assessing sequenced read quality

Read sequence data from each sample were examined for base-call quality using standard software for the purpose, FastQC (www.bioinformatics.babraham.ac.uk/projects/fastqc/). Summaries of the Q-score quality control data accompanying the sequence data from each tissue sample are contained in Dataset 4 (Data Citation 1), with descriptions of the column titles contained in the associated readme file.

#### *De novo* transcriptome assembly quality assessment

To reduce the number of partial or erroneous contigs during transcriptome assembly we used the ‘align and estimate abundance.pl’ script from the Trinity package^34^, to map reads back to the assembly. All contigs with fewer than four reads per million mappable reads were discarded, as previously described^39,40^. Trinity toolkit utilities were then used to assess the quality of resulting transcriptomes. The N50 contig lengths were 2531, 2433, and 2258 bases for liver, skin and spleen assemblies respectively. Similarly, the total number of Trinity ‘genes’ assembled were 21269, 26894, and 28876 for liver, skin and spleen assemblies respectively. The RNA-Seq read representation of our tissue-specific transcriptome assemblies were above the optimal threshold (80%) for proper read pairs (skin 85.24%, liver 89.68% and spleen 83.43%). When considering the representation of protein-coding genes in the assemblies, 65% of skin, 70% of liver and 58% of spleen sequences with BLAST matches had 80% alignment coverage.

#### Functional annotation quality control

The functional annotation software suite BLAST2GO^36^ uses standard algorithms (including the basic linear alignment search tool [BLAST]), as well as quality measures to obtain most parsimonious functional gene annotations. The three most useful measures are (1) the minimum Expect value (E-value), (2) the mean sequence similarity, and (3) consideration of sequence length. In order to exclude ‘chance’ annotations, we employed the commonly accepted thresholds of retaining annotations with a minimum E-value of<1x10^−4^ and mean sequence similarity>60%. These data (and further explanations of these measures) can be found in Dataset 6 (Data Citation 1).

#### Testing for potential batch effects of tissue sampling date on RNA-seq results

**Figure 3:**
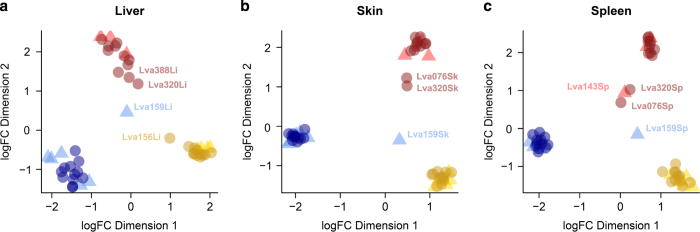
Unsupervised classical (metric) multi-dimensional scaling plots (using plotMDS and recommended settings from the Bioconductor package EdgeR) demonstrating leading log-fold differences in gene expression results between pairs of samples. Uninfected negative control frog samples (▲), Bd-exposed frog samples (●). Samples predominantly cluster by source population (colour groups: Eucumbene—blue, Grey Mare—red, and Kiandra—yellow). (**a**) Liver samples, (**b**) skin samples, and (**c**) spleen samples. Visually identified group outliers have been labeled with frog and tissue ID for ease of comparison (they were not removed from analyses). Reprinted with permission from Grogan et al. (2018)^21^.

In an accompanying study examining differential gene expression^21^ we compared the numbers of differentially expressed genes shared between unexposed control frogs (pooled between populations) sampled at different dates (4, 8 and 14 DPE). Our results demonstrated that there was no evidence for a sampling time batch effect in either the skin or spleen samples, but there was evidence for a mild batch effect in the liver samples (158 genes were significantly differentially expressed between the four and eight day post exposure sampling groups, in both the infected and controls). This difference may have been attributable to relatively rapid liver autolysis compared with the other two tissues sampled (livers were also last to be sampled during necropsy), and a mildly increased sampling time taken during the first sampling session. In the same study^21^ we compared gene expression levels between negative control (uninfected) and exposed (infected) frog groups and as expected, found distinct clustering by population for all three tissue types using multi-dimensional scaling plots (Fig. 3, reprinted with permission from Grogan et al. 2018^21^).

### Quality control of the metabolomics study

#### Exogenous analytical standards for quality control

To allow the collection of retention-time aligned data, consistent with the metabolite reference MS-library, the GC inlet pressure was adjusted prior to sample analysis to elute a mannitol (D-mannitol, 6TMS) external reference standard at a retention time of 30.6 min, and the ^13^C-sorbitol (^13^C-sorbitol, 6TMS) internal reference standard at 30.7 min. Prior to GC-MS acquisition, to each sample was added five microliters of hexane containing a mixture of eight n-alkane retention index reference standards, to allow retention index (RI) calculation. A RI was calculated for each analyte measurement relative to the RI of each of the eight n-alkanes. Metabolite identities were assigned by comparison to a MS-library of authentic metabolite standards analysed under the same analytical conditions, with the RT of mannitol (6TMS) fixed as described, and metabolite match criteria requiring a RI of±3 (Data Citation 3), (Dataset 9; Data Citation 1).

#### Supportive data for metabolite identification

Metabolite identities were assigned by comparison to a MS-library of authentic metabolite standards, as already described. Where an additional confirmation of metabolite identity was sought, for example in the identification of serotonin (for which the mass spectrum is predominantly dominated by a single ion, m/z 174, together with the TMS-derivatives of other biogenic amines), pooled replicates of the metabolite extracts were set aside for verification of metabolite identification by ‘spiking’ with a series of metabolite standards, prior to GC-MS analysis. Identity was confirmed by a corresponding increase in intensity of the respective formerly observed ion(s) within the mass spectra. A single reference pool of metabolite extract was divided into six aliquots. Prior to drying, 65 μl of 25 μg mL^−1^ of a series of metabolite reference standards (in 50% methanol) was added to three replicates, and 65 μl of 50% methanol was added to another three replicate tubes for comparison. Each was dried in preparation for GC-MS analysis.

#### Exclusion of analytical artifacts

Analytical artifacts were determined by analysis of blank extraction controls (containing no tissue sample). Analytes observed within these analyses were removed from the metabolite measurement data, as they were deemed of non-biological origin and thus not metabolites. Analytes suspected of being analytical artefacts (by MS-library match) but not confirmed by the extraction controls were not removed from the final data matrix (marked as likely artifacts within Dataset 9).

#### Testing for potential instrumental batch effects on metabolomics results

**Figure 4:**
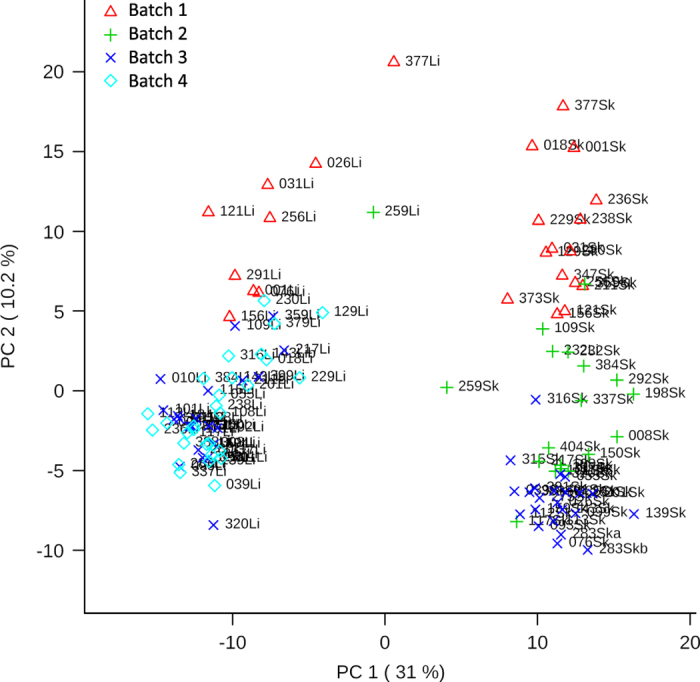
Principal components analysis scores plot for metabolite expression from both liver and skin tissue samples (L. Grogan unpublished data). Expression data was first normalized by Total Area under Chromatogram by sample, and overall dataset log transformation and pareto scaling. Samples separate by tissue type on the horizontal axis (Principal Component 1, representing 31% of the variability) with liver samples on the left (‘Li’ in sample name) and skin samples on the right (‘Sk’ in sample name). Samples separate only mildly by batch (date of processing) on the vertical axis (Principal Component 2, representing 10.2% of the variability), consistent with preventative GC-MS maintenance regimens.

In an accompanying study examining differential metabolite accumulation (L. Grogan unpublished data) we compared the sum of metabolite concentrations between metabolite extraction dates and found mild instrumental batch effects in the skin tissue samples, consistent with preventative GC-MS maintenance regimens (Fig. 4).

## Additional information

**How to cite**: Grogan, L. F. *et al.* Survival, gene and metabolite responses of *Litoria verreauxii alpina* frogs to fungal disease chytridiomycosis. *Sci. Data* 5:180033 doi: 10.1038/sdata.2018.33 (2018).

**Publisher’s note**: Springer Nature remains neutral with regard to jurisdictional claims in published maps and institutional affiliations.

## Supplementary Material



Supplementary File 1

## Figures and Tables

**Table 1:** Experimental design for *L. v. alpina* clinical survival experiment, Experiment A.

**Population (total number frogs)** *	**Clutch (total number frogs)** ^*^	**Exposure group** ^**†**^	**Total number of frogs**	**Number of males** ^**‡**^	**Number of females** ^**‡**^	**Number with undetermined gender**
Eucumbene (99)	A (30)	E	20	7	13	0
		C	10	6	4	0
	B (19)	E	19	10	9	0
		C	0	0	0	0
	C (29)	E	20	10	10	0
		C	9	4	5	0
	D (21)	E	19	11	8	0
		C	2^§^	0	1	1
Grey Mare (80)	A (14)	E	14	7	6	1
		C	0	0	0	0
	B (26)	E	20	10	10	0
		C	6	3	3	0
	C (29)	E	20	11	9	0
		C	9	6	3	0
	D (11)	E	11	9	2	0
		C	0	0	0	0
Kiandra (100)	A (25)	E	20	9	11	0
		C	5	1	4	0
	B (25)	E	20	12	8	0
		C	5	1	4	0
	C (25)	E	20^|^	8	10	2
		C	5	3	2	0
	D (25)	E	20	11	9	0
		C	5	1	4	0
Ogilvies (76)	A (19)	E	19	13	6	0
		C	0	0	0	0
	B (40)	E	20	7	13	0
		C	20	6	14	0
	C (17)	E	16	9	7	0
		C	1^¶^	0	0	1
Numbers of *L. v. alpina* frogs from each population and clutch are outlined, including details of blind randomized block design used for allocation of treatment groups (exposed frogs versus sham-exposed negative control frogs).						

*Total number of frogs in parentheses for each group. † E represents exposed frogs, C represents control frogs. ^‡^Gender as determined by post-mortem coelomic examination. ^§^Includes one frog that died pre-exposure and was excluded from data records. ^|^This number includes a frog that died of anasarca post-exposure, unrelated to chytridiomycosis, data still included (frog ID: Lva162). ^¶^This frog died pre-exposure and was excluded from data records.

**Table 2:** Experimental design for *L. v. alpina* tissue response experiment, Experiment B.

Populations	Exposure – Day 0	Day 4	Day 8	Day 14
	Total # exposed	# exposed sampled	# exposed sampled	# exposed sampled
	(total # control)*	(# control sampled)^†^	(# control sampled)^†^	(# control sampled)^†^
Grey Mare (clutch B)	12 (3)	4 (1)	4 (1)	4 (1)
Eucumbene (clutch D)	12 (6)	4 (2)	4 (2)	4 (2)
Kiandra (clutch B)	12 (6)	4 (2)	4 (2)	4 (2)
Total	36 (15)	12 (5)	12 (5)	12 (5)
Numbers of frogs from each population and treatment group (Bd exposed or unexposed control) sampled at each time point post exposure are indicated. The 10 frogs from Experiment A that demonstrated clinical signs are excluded from this table (as their sampling date was contingent on infection progression). *Total number of unexposed control frogs shown in parentheses ^†^Number of unexposed control frogs sampled shown in parentheses.				

**Table 3:** Experimental design for *L. v. alpina* clinical survival experiment, Experiment A

**Dataset Number**	**Data Citation Number**	**Description**	**Number of files**	**File names**	**File formats**	**Readme file**	**Description of Readme file**
1	1	Animal signalment, survival and pathogen infection intensity data from the animals in Experiment A	1	Dataset 1 Experiment A results data	.csv	Yes	Column header descriptions
2	1	Animal signalment and pathogen infection intensity data from the animals in Experiment B	1	Dataset 2 Experiment B results data	.csv	Yes	Column header descriptions
3	2	Raw RNA-Seq reads from skin, liver and spleen tissues of each frog sampled as part of Experiment B	362	Please refer to NCBI records (NCBI BioProject PRJNA356986)	.fastq.gz	No	N/A
4	1	Summaries of the phred Q-score quality control data accompanying the sequence data from each tissue sample (in Dataset 3)	1	Dataset 4 Mean FastQC Quality scores	.csv	Yes	Column header descriptions
5	1	*De novo* assembled transcriptomes for each tissue type from frogs in Experiment B	6	Dataset 5.1 Liver-filtered-condensed	.fasta	Yes	Sequence identifier line descriptions for both nucleotide and amino acid sequences
				Dataset 5.2 Liver-filtered-condensed-transdecoder			
				Dataset 5.3 Skin-filtered-condensed			
				Dataset 5.4 Skin-filtered-condensed-transdecoder			
				Dataset 5.5 Spleen-filtered-condensed			
				Dataset 5.6 Spleen-filtered-condensed-transdecoder			
6	1	Functional annotation data (from the Gene Ontology [GO] consortium, Enzyme Code and Inter Pro databases) for each gene assembled, across the three tissue types (skin, liver and spleen) from frogs in Experiment B	3	Dataset 6.1 Liver transcriptome functionally annotated	.csv	Yes	Column header descriptions
				Dataset 6.2 Skin transcriptome functionally annotated			
				Dataset 6.3 Spleen transcriptome functionally annotated			
7	1	Raw gene/transcript counts data for each frog tissue sample from Experiment B resulting from allocation of sequence reads to genes from the tissue-specific transcriptome assemblies	12	Dataset 7.1 Liver gene expression matrix.isoforms.counts	.txt	No	N/A
				Dataset 7.2 Liver gene expression matrix.isoforms.TMM.EXPR			
				Dataset 7.3 Liver gene expression matrix.isoforms.TPM.not_cross_norm			
				Dataset 7.4 Liver gene expression matrix.isoforms.TPM.not_cross_norm.TMM_info			
				Dataset 7.5 Skin gene expression matrix.isoforms.counts			
				Dataset 7.6 Skin gene expression matrix.isoforms.TMM.EXPR			
				Dataset 7.7 Skin gene expression matrix.isoforms.TPM.not_cross_norm			
				Dataset 7.8 Skin gene expression matrix.isoforms.TPM.not_cross_norm.TMM_info			
				Dataset 7.9 Spleen gene expression matrix.isoforms.counts			
				Dataset 7.10 Spleen gene expression matrix.isoforms.TMM.EXPR			
				Dataset 7.11 Spleen gene expression matrix.isoforms.TPM.not_cross_norm			
				Dataset 7.12 Spleen gene expression matrix.isoforms.TPM.not_cross_norm.TMM_info			
8	3	Metabolite accumulation data from skin and liver tissues for each frog from Experiment B	N/A	Please refer to MetaboLights records (MetaboLights MTBLS457)	N/A	No	N/A
9	1	Metabolite accumulation data from skin and liver tissues for each frog from Experiment B	1	Dataset 9 Metabolomics results data	.csv	Yes	Column header descriptions and associated analyte data
Numbers of *L. v. alpina* frogs from each population and clutch are outlined, including details of blind randomized block design used for allocation of treatment groups (exposed frogs versus sham-exposed negative control frogs).							
